# Influence of Antenatal Education on Birth Outcomes: A Systematic Review Focusing on Primiparous Women

**DOI:** 10.7759/cureus.64508

**Published:** 2024-07-14

**Authors:** Anna-Maria Athinaidou, Eirini Vounatsou, Ilianna Pappa, Vikentia C Harizopoulou, Antigoni Sarantaki

**Affiliations:** 1 Department of Midwifery, University of West Attica, Athens, GRC; 2 Department of Midwifery, Faculty of Health and Social Sciences, University of Bedfordshire, Bedford, GBR; 3 1st Department of Obstetrics and Gynecology, Papageorgiou General Hospital, Medical School, Aristotle University of Thessaloniki, Thessaloniki, GRC

**Keywords:** vaginal birth, delivery mode, fear of childbirth, postpartum depression, childbirth self-efficacy, maternal experiences, antenatal classes, prenatal education

## Abstract

The primary objective of prenatal education programs is to furnish expectant mothers with the necessary knowledge for childbirth and early parenting. Despite the extensive implementation of these programs, the efficacy of these interventions remains unclear. This systematic review endeavored to consolidate the extant evidence pertaining to the effects of prenatal education on birth outcomes and experiences.

Fourteen studies, comprising various study designs executed across diverse countries, were incorporated in this review. The outcomes assessed in these studies encompassed fear and anxiety, pain, delivery mode, interventions, postpartum depression, and self-efficacy. Additionally, the risk of bias and study limitations were also summarized.

The results manifested that prenatal education was effective in diminishing the fear and anxiety associated with childbirth and enhancing self-efficacy in the majority of the studies. Moreover, several studies found that engaging in prenatal education augmented the preference for unmedicated vaginal birth. However, the effects of prenatal education on postpartum outcomes were less consistent. The primary constraints of the included studies were their minute sample sizes and brief follow-up periods.

Nonetheless, the existing evidence proposes that prenatal education is beneficial for first-time mothers in terms of alleviating anxiety and augmenting agency during delivery. Prenatal education can equip pregnant individuals with the necessary knowledge and skills to navigate the perinatal period successfully. Further research is requisite to identify the optimal practices for diverse populations on a global scale.

## Introduction and background

Childbirth is a seminal event in a woman's life that can have lasting effects on her well-being and future reproductive choices. The way she remembers and interprets this experience can have a profound impact on her life [1]. Numerous women possess a favorable outlook on their labor and feel empowered by the hurdles they conquer. Nevertheless, certain laboring women characterize their experience as a demanding one that is fraught with anxiety, helplessness, and dissatisfaction [2,3]. Managing women's expectations through prenatal education is crucial for fostering a realistic understanding of the labor process. By doing so, women may experience greater control and capacity to tolerate pain, which may ultimately lead to increased satisfaction and cherished recollections [4].

Numerous research studies have investigated the relationship between prenatal education and women's perceptions of labor. According to previous research conducted by midwives, education on childbirth has been shown to lead to more favorable attitudes and feelings of control during labor [5]. Additional studies have shown that the attitudes and social support experienced in prenatal classes have a significant impact on women's perceptions of pain [6]. Expert counsel recommends that childbirth educators focus on differentiating between self-management (managing breathing and responding to discomfort) and situational management (labor routines) [7]. Given that many first-time mothers often report being unprepared for the level of pain they experience during childbirth, it is crucial to have realistic discussions about pain management during prenatal education [8]. As experts in pregnancy, labor, and delivery, midwives are well-positioned to provide comprehensive, professionally guided prenatal education to ensure that mothers-to-be are adequately prepared for the challenges ahead [9-10].

Although prenatal education is not always practiced in certain parts of the world, it holds great importance in the prenatal care of expectant mothers in the majority of Western countries [11]. Prenatal education serves the dual purposes of acquainting pregnant parents with the key components of pregnancy and childbirth as well as preparing the mother and/or spouse for the care of the newborn. The extent of prenatal education across different countries is not well-documented. However, for first-time mothers in Australia, the attendance rate is reported to be 84% [12], while in Canada, it is estimated to be approximately 33% [13]. Prenatal education has typically been imparted through experiences shared with family or relatives, particularly in low-income, rural, or uneducated populations, as well as through the internet or shared media groups, which cater to younger, more media-dependent individuals [14]. However, the reliability and usefulness of such educational techniques during pregnancy, as well as the manner in which the information is delivered and the quality of the content, are not consistently dependable or effective [14].

This context explains the current study's goal, which is to investigate the connection between pregnant individuals' attendance at prenatal classes and the labor experiences that women perceive. The process of labor can be a simultaneously stressful and empowering experience. Women's memories and choices regarding childbearing may be influenced by preconceived notions and feelings of control. Prenatal education presents a valuable opportunity to equip expectant mothers with practical knowledge and coping mechanisms, as well as realistic expectations for the childbirth experience. This study aims to evaluate whether comprehensive prenatal education, particularly when provided by skilled midwives, can positively impact the attitudes of parturients. The findings could encourage maternity care professionals to concentrate their recruitment efforts on prenatal education attendance. Improving labor satisfaction and control for women by providing proper education is still a crucial endeavor that affects the overall well-being of mothers.

## Review

Methods

Search Strategy

The systematic review was carried out using the Preferred Reporting Items for Systematic Reviews and Meta Analyses (PRISMA) statement [15]. The main goal of the article was to compare the experiences of first-time mothers regarding childbirth, those who received prenatal education, and those who did not. Utilizing the following PICO framework in our systematic review, we meticulously pinpointed and elucidated the particular aspects of the research question, guaranteeing that the review was methodical, focused, and comprehensive. This approach allowed for the efficient discovery of pertinent studies, the precise selection of studies for the review, and the cohesive examination of results across multiple studies.

P (population): This refers to the patient or population group being considered. In the case of our review, this was "primiparous women" (first-time mothers).

I (intervention): This is the primary intervention being studied. For our systematic review, the intervention was "prenatal education," including more specifically programs designed to prepare women for childbirth.

C (comparison): The comparison usually involves the current standard of care or a different type of intervention against which the primary intervention is evaluated. In our review, this could be "no education," "standard prenatal care without additional education," or "different types of educational interventions."

O (outcomes): These are the results or effects of interest that are measured to determine the intervention’s effectiveness. For our review, relevant outcomes could include "birth outcomes," which might encompass measures like labor duration, use of pain relief during labor, maternal confidence in childbirth, rates of cesarean delivery, or neonatal outcomes.

The study population consisted of second and third-trimester pregnant women. The intervention was attending thorough prenatal education sessions, particularly those led by midwives. The comparison group comprised pregnant women who did not participate in official prenatal education. The outcomes were the women's opinions and satisfaction with their labor and delivery experience, taking into account factors such as fulfillment, pain management, and control. Validated survey tools were used to collect data, which were then compared between the participants in prenatal classes and non-participants in the early postpartum period.

Database Search

**Figure 1:**
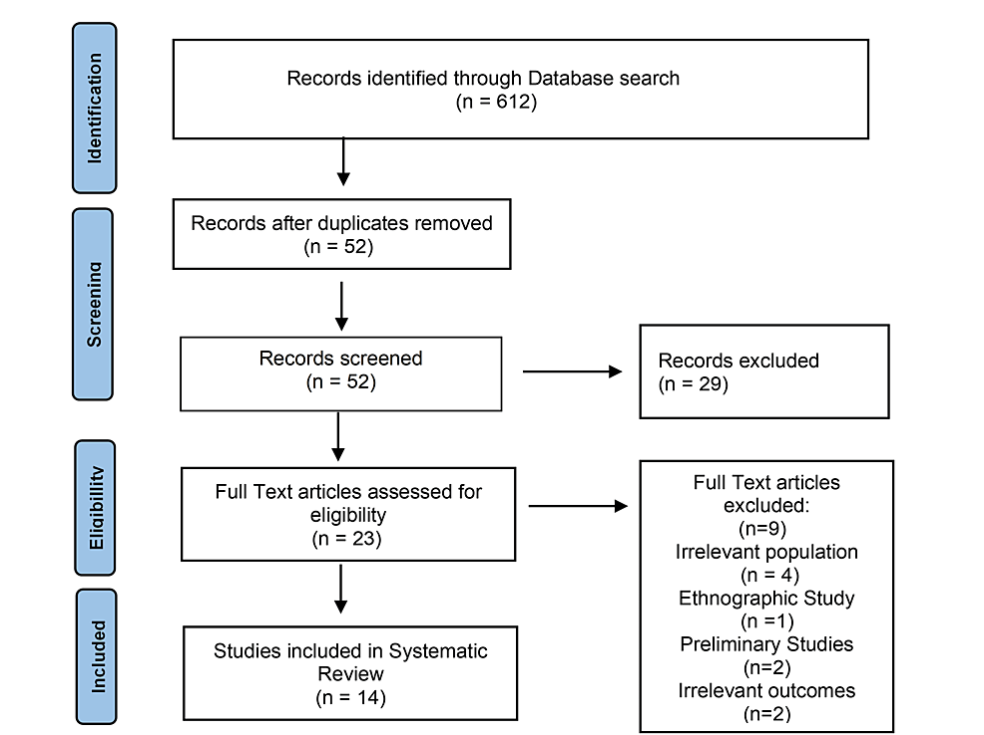
The Preferred Reporting Items for Systematic Reviews and Meta-Analyses (PRISMA) diagram for the selection of the included studies

The search for relevant studies was conducted throughout October 2023, utilizing the scientific literature databases of Cochrane Library, PubMed, the Cumulated Index to Nursing and Allied Health Literature (CINAHL), and the Excerpta Medica Database (EMBASE). The search was executed using specific keywords, including (prenatal education OR prenatal class* OR childbirth education OR Lamaze) AND (labor OR labour) AND (perception OR experience OR attitude OR satisfaction) AND (birth OR childbirth OR intrapartum OR delivery) AND (no prenatal education OR no prenatal classes OR no childbirth education OR no Lamaze OR unprepared OR untrained OR uninformed OR uneducated OR uninstructed OR unpreparedness OR lack of preparation OR absence of prenatal education OR did not attend prenatal classes OR did not receive prenatal education OR no formal prenatal instruction OR no prenatal knowledge OR naive OR unaware OR inexperienced). In this systematic review, the PRISMA statement for reporting systematic reviews that is proposed by the Cochrane Collaboration was followed (Figure 1).

Eligibility Criteria

The following types of studies were included in the search: retrospective research, population-based studies, cross-sectional studies, cohort studies, case-control studies, and randomized or non-randomized controlled trials. The inclusion criteria for the studies were as follows: (1) conducted on female subjects; (2) controlled groups assessing the structured prenatal education classes offered to expectant parents; (3) observational studies presenting the outcome of a structured intervention; (4) studies examining the notion of prenatal education; (5) studies that included a control group that received no education or sham/placebo education; (6) studies that compared the control and experimental groups using the same outcome measures; (7) relevant English-language publications; and (8) research involving more than twenty participants. Review articles, editorials, comments, letters, abstract-only publications, case studies, meta-analyses, and systematic reviews were excluded from this systematic review.

Inclusion Criteria

The inclusion criteria for the systematic review were consistent across several studies. Specifically, the participants were limited to nulliparous or primiparous women, as first-time mothers would presumably benefit more from prenatal education. The gestational age of the participants was set between 20 and 36 weeks to include women in the second and third trimesters of pregnancy. All studies established an age criterion of eighteen years or older to control for the potential confounding factor of a very young maternal age. Additionally, some studies required participants to possess a minimum level of literacy in the local language to enable them to attend prenatal classes and complete surveys.

Results and discussion

Study Characteristics

Table 1 displays the primary characteristics of the studies that were incorporated into this systematic review. The methodological quality of these studies was high, as they all possessed clear objectives and a detailed description of their study design, statistical analysis, and outcomes. The inclusion criteria for the populations of pregnant women varied among the studies. Of these studies, five were randomized controlled trials [16-20], one was a quasi-experimental study [21], two were randomized clinical trials [22,23], two were prospective studies [24, 25], two were cross-sectional surveys [26,27], one was a comparative study [28], and one had a nonrandomized post-test design [29].

**Table 1:** Key characteristics of the included studies *Microsoft Corporation, Redmond, Washington, United States NA: not available; PECs: prenatal education classes; FOC: fear of childbirth; CPC: childbirth preparation course

Author	Year	Country	Journal	Sample size	Study design	Inclusion criteria	Primary outcome	Secondary outcome	Results/conclusions
Bahrami et al. [16]	2013	Iran	Int J Fertil Steril	80 women in the intervention group and 80 women in the control group	Single-blind randomized controlled trial	First-time pregnant women with a singleton fetus were invited to participate in the study if they met the following criteria: i. aged 18 to 35, ii. between 24- and 28-weeks’ gestation, iii. without a history of medical, psychological, and infertility diseases, iv. with at least eight prenatal visits during pregnancy, whilst two visits occurred before the 20th week of pregnancy, v. reading and writing in Farsi (Persian), and vi. giving written informed consent	Quality of life	NA	The study showed that women receiving prenatal education had higher levels of happiness and satisfaction in their overall quality of life and health, respectively
Uludag et al. [17]	2022	Turkey	Midwifery	23 pregnant women in the intervention group and 23 in the control group	Single-blind randomized controlled trial	Age of 18 years or more, gestation of 24–34 weeks, graduation at least from primary school, nulliparity, not being at high risk in pregnancy, ability to use the application of Microsoft Teams*, not having a psychiatric disease, and not having attended any other antenatal programs in the antenatal period.	The Oxford Worries about Labour Scale, the Fear of Birth Scale, the Prenatal Self Evaluation Questionnaire, and the Fear of COVID-19 Scale	NA	Online prenatal education decreased worries about labor, fear of childbirth, and fear of COVID-19, and improved preparedness for labor. Online prenatal education offered during the COVID-19 pandemic is effective in preparedness for labor.
Cankaya et al. [18]	2021	Turkey	Clin Nurs Res	120 primiparous pregnant women, with 60 women assigned to the prenatal education group and 60 women assigned to the control group	Pre- and post-test randomized controlled study	Nulliparous women older than 18 years of age with a healthy singleton pregnancy with 20 and 32 weeks of gestation were included. Participation criteria also include childbirth at full term, having a healthy newborn (born at 38–42 weeks), and not participating in another prenatal program	Birth fear, depression, anxiety, stress, childbirth self-efficacy, and mode of delivery	Postnatal birth fear, depression, anxiety, and stress symptoms	The study found that prenatal education had significant clinical benefits for pregnant women, reducing birth fear, depression, anxiety, and stress symptoms, and increasing childbirth self-efficacy. Additionally, the prenatal education group had a higher rate of vaginal births compared to the control group. Based on these findings, the authors concluded that all pregnant women should receive antenatal education.
Dai et al. [19]	2021	China	Patient Educ Couns	76 participants who were randomly assigned to either the intervention group (n=38) or the control group (n=38)	Randomized controlled trial	Primipara, singleton pregnancy, aged 20-35 years, gestational age of 24-32 weeks, having an FOC (as identified by a score of > 66 on the Chinese version 93 of the Wijma Delivery Expectancy/Experience Questionnaire 94 (WDEQ-A))	Effectiveness of simulation-based childbirth education (SBCE) on fear of childbirth.	NA	Scores on the WDEQ-A of the intervention group were statistically lower than those of the control group (p < 0.05). The Childbirth Self-Efficacy Inventory (CBSEI) scores of the intervention group were significantly higher than those of the control group (P < 0.05). The cesarean birth rate of the intervention group was lower than that of the control group (34.61% vs. 46.67%, p > 0.05). Simulation-based childbirth education alleviates the FOC, increases childbirth self-efficacy, and improves birth outcomes, providing a new perspective to alleviate the FOC of primiparas in the future.
Masoumi et al. [20]	2016	Iran	J Family Reprod Health	150 pregnant women randomly allocated to control (n=75) and intervention groups (n=75)	Randomized control trial	Single fetus, no chronic diseases such as diabetes, heart and lung chronic diseases, no infertility, no high-risk pregnancy, no history of psychiatrist visit, not using specific drugs, and gestational age of 20 weeks	Fear of natural childbirth	NA	Fear score in group A compared to group B was significantly reduced (51.7 ± 22.4 vs. 58.7 ± 21.7) (p = 0.007). Physiologic delivery was the first choice of type of childbirth after training in pregnant women in group A (58.7%). Educational programs could serve as an important tool in reducing women's fear of natural childbirth and in choosing physiologic birth. Including physicians and midwives is crucial for the delivery process, which encompasses aspects of physiology, education, and counseling for pregnant women.
Sercekus et al. [21]	2016	Turkey	Midwifery	35 couples in the intervention group and 37 couples in the control group	Quasi-experimental study	Gestation of 26-28 weeks, minimum education level of primary school graduation, nulliparous, not at high risk in pregnancy, and not did not attend any other antenatal program in the antenatal period. Additionally, inclusion required giving birth at full term, having a healthy newborn, and having experienced no postnatal complications	Total fear of childbirth	NA	Prenatal education was found to reduce the FOC and increase childbirth-related maternal self-efficacy. However, prenatal education had no effect on parental attachment. It is recommended that widespread prenatal education programs should be provided in developing countries, and the content of the education program about parental attachment should be increased.
Beydokhti et al. [22]	2021	Iran	Nurs Open	137 pregnant women (66 to the intervention group and 71 to the control group)	Randomized clinical trial	Women in 30–35 weeks of pregnancy, no history of depression, reading and writing literacy, a healthy fetus in ultrasonography, living in the city, and having given consent for participating in the study	Predisposing, reinforcing, enabling factors and post-partum depression	NA	Independent t-test showed a significant difference between the two groups in terms of the mean score of predisposing, reinforcing, enabling factors and post-partum depression (p < .05). Regression tests indicated predisposing, reinforcing, enabling factors and general health as the most important factors associated with post-partum depression (p < .05). The results supported the effectiveness of the educational intervention on reducing post-partum depression and showed that implementing these training during pregnancy leads to a reduced level of post-partum depression.
Firouzbakht et al. [23]	2015	Iran	Ann Med Health Sci Res	195 pregnant women, study group (n=132) and control group (n= 63)	Randomized clinical trial	Pregnant women attending health centers in Amol, Iran, from 20 weeks of gestation age	Anxiety levels, pain intensity during childbirth, and intervention during labor (such as episiotomy and cesarean section)	NA	The study found that pregnant women with a high level of education were more interested in participating in prenatal education classes. The case group (who received prenatal education) had lower anxiety levels and pain intensity during childbirth compared to the control group.
Soriano-Vidal et al. [24]	2018	Spain	Midwifery	212 pregnant women of which 78.3% were nulliparous	Multicenter, observational, prospective study	Participation in at least five of the eight planned PECs directly related to the birth plan (pregnancy, childbirth, puerperium, newborn care, and breastfeeding), and the ability to speak, write, and read Spanish	The influence of prenatal educational classes led by midwives on women's birth preferences.	NA	Significant differences in birth plan preferences were observed before and after the prenatal classes, with three items showing an increase: the ability to push spontaneously, episiotomy avoidance, and early breastfeeding.
Balasoiu et al. [25]	2021	Romania	Medicina (Kaunas)	89 pregnant women of which 62 participated in prenatal education classes and 27 were in the control group	Prospective study	Pregnant women aged over 18 who gave birth at Bucur Maternity, Saint John Hospital, Bucharest, Romania	Newborn care, newborn feeding, breastfeeding, birth preparation, partner’s support, mother alimentation, and hygiene	NA	The benefits of prenatal education were recognized by women who attended the prenatal lecture, while women who did not participate underestimated the utility of the topics.
Shi et al. [26]	2015	China	Environ Health Prev Med	604 mothers	Cross-sectional survey	Mothers aged 20–45 years who had given birth (by natural delivery or cesarean section) between 1 May 2011 and 1 May 2012	Five main outcome measures: personal information, their prenatal examination utilization, delivery mode, recovery status from the delivery, and the participation, implementation, and effect of the prenatal education curriculum	NA	The mothers who participated in prenatal education classes provided by hospitals during their latest pregnancy had a higher rate of attending all the required prenatal examinations and a higher rate of recovering very well, than those who did not participate in prenatal education classes. However, there was no statistical difference in the delivery mode between mothers who participated and those who did not.
Yohai et al. [27]	2018	Israel	J Perinat Med	53 primiparous women who attended prenatal childbirth preparation course and 54 primiparous women who did not	Cross-sectional study	Women with full oral and written comprehension of the Hebrew language, between the ages of 18 and 46, following their first delivery at Soroka University Medical Center (SUMC), Beersheba, Israel	The State-Trait Anxiety Inventory (STAI)	NA	The STAI score was significantly lower in the study group compared with controls (p = 0.025). The knowledge acquired in the CPC has positive effects on the course of labor and delivery outcomes and leads to higher rates of breastfeeding.
Mueller et al. [28]	2020	Alaska	J Perinat Educ	82 attendees of childbirth classes and 115 non-attendees of childbirth classes	Comparative study	Low-risk, primiparous women	Type of birth, second stage intervention, prostaglandin, pitocin, active range of motion (AROM), epidural, and progression at admission	NA	Labor interventions were used significantly less in women who took a childbirth class. Childbirth education may help women prepare for what to expect during childbirth and minimize the use of medical interventions.
Gürkan et al. [29]	2017	Turkey	Clin Exp Health Sci	65 participants, with 31 participants in the education group and 34 participants in the control group.	Nonrandomized, post-test–control group design	Aged over 18 years, in the 20th-36th gestational weeks, primipara, married, without pregnancy-related complications (such as preeclampsia or diabetes), and not diagnosed with depression in the pre-pregnancy period	Postpartum functional status at six weeks and six months, as assessed using the Doğum Sonrası Fonksiyonel Durum Envanteri (DS-FDE)	Postpartum depression status at six weeks and six months, as assessed using the Edinburgh Postnatal Depression Scale (EPDS)	The study found that there was no significant difference in postpartum functional status scores and postpartum depression scores between the education group and the control group at both six weeks and six months. Therefore, the study concluded that prenatal education may not be effective in improving postpartum functional status and reducing postpartum depression.

Risk of Bias Assessment

The assessment of the risk of bias in the studies included in this review was conducted using the Risk of Bias in Non-randomized Studies - of Interventions (ROBINS-I) tool [30]. The same is presented in Table 2.

**Table 2:** Risk of bias in the studies included the review Domains: D1: Bias due to confounding; D2: Bias in selection of participants into the study; D3: Bias in classification of interventions; D4: Bias due to deviations from intended interventions; D5: Bias due to missing data; D6: Bias in the measurement of outcomes

Study	D1	D2	D3	D4	D5	D6	Overall
Bahrami et al. [16]	Low	Low	Low	Medium	Low	Low	Low
Uladag et al. [17]	Low	Unclear	High	Unclear	Low	Medium	Medium
Çankaya et al. [18]	Low	High	Low	Low	Low	High	Medium
Dai et al. [19]	Low	Unclear	Low	Low	Unclear	Medium	Low
Masoumi et al. [20]	Low	High	Low	Low	Unclear	Low	Medium
Sercekus et al. [21]	High	High	Low	Unclear	Low	Low	High
Beydokhti et al. [22]	Low	Unclear	Low	Low	Unclear	Low	Low
Firouzbakht et al. [23]	High	High	Low	Low	Unclear	Medium	Medium
Soriano et al. [24]	High	Medium	Low	Medium	High	Medium	High
Balasoiu et al. [25]	High	Not applicable	Low	Not applicable	Not applicable	Low	Unclear
Shi et al. [26]	Not applicable	Not applicable	Low	Low	High	Medium	Low
Yohai et al. [27]	High	High	High	Unclear	Low	Medium	High
Mueller et al. [28]	Not applicable	Not applicable	Not applicable	Unclear	Unclear	Unclear	Unclear
Gurkan et al. [29]	High	High	Low	High	Unclear	Medium	High

Outcomes

The included studies demonstrated considerable variability in the outcome measures employed. Common outcome measures comprised anxiety, fear of childbirth/labor, and childbirth self-efficacy as well as the mode of delivery and postpartum depression. The outcomes of five studies have consistently shown that prenatal education proves to be effective in reducing the levels of fear and anxiety associated with childbirth and can enhance the self-efficacy of first-time mothers in relation to childbirth [16-22]. Prenatal education has been found to have an impact on birth preferences and outcomes. Studies have shown that it increases the preference for and rates of spontaneous vaginal birth as compared to cesarean deliveries and leads to a reduction in interventions during birth, such as episiotomy and epidural anesthesia [23-27]. Although the outcomes of the studies exploring the impact of prenatal education on postpartum results were mixed, certain researchers suggested that prenatal education had no bearing on postpartum depression or functional capacity, while others revealed that prenatal education was associated with increased breastfeeding rates and improved newborn care knowledge [28,29]. In general, the primary advantages were found to be a decrease in apprehension and uncertainty related to childbirth, along with an increase in self-assurance and control for first-time mothers.

Discussion

Prenatal education is implemented through various channels such as classes, workshops, online resources, and one-on-one sessions with maternity healthcare providers. Different countries have their own approaches to prenatal education, tailored to their cultural practices and healthcare systems [31]. For example, some countries integrate traditional practices and beliefs into their prenatal education programs, acknowledging the influence of cultural customs on pregnancy and childbirth. In contrast, other nations may focus more on evidence-based approaches and technological advancements in prenatal care [32]. Despite these differences, the underlying goal remains consistent: to ensure that expectant parents have access to accurate information, support, and resources to navigate the journey of pregnancy and childbirth. This concerted effort in prenatal education contributes to improved maternal and infant health outcomes on a global scale [33-37]. 

Prenatal education extends beyond simply imparting fundamental knowledge; it also fosters a supportive community among expectant parents, healthcare providers, and others, proving particularly beneficial for first-time parents through shared experiences and emotional support. [38]. This educational approach not only informs but also empowers parents, encouraging active participation in prenatal care and childbirth planning, which boosts their confidence and sense of ownership in the process. The prenatal care model that focuses on parental education incorporates cultural and societal elements that affect prenatal care by integrating various practices and beliefs, thereby fostering inclusivity. Despite the differences in prenatal education methods across various countries, influenced by unique cultural norms and healthcare systems, the ultimate goal is to provide expectant parents with the essential knowledge and support needed to enhance the health and well-being of the mother-infant dyad worldwide [39-42].

This study aimed to investigate the impact of prenatal educational programs on the perceptions of first-time mothers regarding birth and delivery. Several studies have demonstrated that a substantial portion of first-time mothers experience fear, anxiety, and emotional distress regarding the impending labor process [43,44]. However, the extent to which prenatal education alters these perceptions has not been thoroughly explored. Although some research has examined the consequences of prenatal interventions on postpartum outcomes [45,46], the effect of prenatal education on perceptions of birth and delivery remains unclear.

Our study identified fear of childbirth and anxiety levels as the most prominent factors affected by these programs, followed by childbirth self-efficacy and delivery mode preferences. These findings are consistent with prior research. In a systematic review and meta-analysis by Demirci et al., which comprised seven articles, the results demonstrated that prenatal education had statistically significant positive effects on the outcome expectancy of labor [47]. Furthermore, in a study conducted by Leutenger et al., the effects of two specific modalities included in prenatal educational programs were investigated. Specifically, the authors assessed the effects of relaxation and breathing techniques on the mother's satisfaction in labor and delivery, the need for pharmacologic support for pain management, and the pain level, as well as the delivery method. The results showed that the techniques positively impacted the mother's self-efficacy and the need for medical interventions such as epidural anesthesia, but had no effects on outcomes related to the neonate. The authors concluded that women who participated in these classes benefited from these prenatal education techniques [48].

In the meta-analysis conducted by Alizadeh-Dibazari et al., a total of 18 studies were examined to evaluate the impact of prenatal education on pain intensity, fear of childbirth, postpartum psychological stress, and overall childbirth experience. The study's findings indicated that prenatal education was effective in reducing fear of childbirth, labor pain, and the likelihood of postpartum depression. However, the authors noted that there was significant inconsistency among the included studies, which impeded the performance of statistical analysis on all parameters [49]. Prenatal counseling and education were found to help mitigate maternal anxiety by providing knowledge and developing coping strategies [50]. 

The primary limitation of our study was the heterogeneity of the study design, outcome measures, timing and duration of prenatal education, and the specific populations targeted, which may have impacted the generalizability of the findings. The dearth of standardization in the studies conducted thus far, which vary in terms of study design, population, and intervention, hampers the ability to make direct comparisons and identify consistent patterns regarding the most effective aspects of prenatal education. Moreover, this standardization deficit is also evident in the published literature [49]. Additionally, there is the potential for self-selection bias and limited generalizability, as many studies had strict inclusion criteria, confining participation to low-risk pregnancies only. Women who opt to participate in prenatal education may differ in substantial ways from those who do not. Consequently, larger, population-representative studies are necessary to elucidate the most effective strategies for prenatal educational interventions across various settings

## Conclusions

This systematic review highlights the significant benefits of prenatal education programs for first-time mothers. These programs effectively reduce fear and anxiety associated with childbirth and enhance maternal self-efficacy. Furthermore, participation in prenatal education increases the preference for unmedicated vaginal births. However, the impact on postpartum outcomes, such as depression and functional status, remains inconsistent. The implementation of prenatal education differs worldwide, shaped by cultural and healthcare system variations. Future research should focus on larger, diverse populations, standardized interventions, and long-term follow-up to identify the most effective prenatal education practices and optimize content and delivery methods for various socioeconomic backgrounds.
